# Regeneration of joint surface defects by transplantation of allogeneic cartilage: application of iPS cell-derived cartilage and immunogenicity

**DOI:** 10.1186/s41232-023-00307-0

**Published:** 2023-11-14

**Authors:** Kengo Abe, Noriyuki Tsumaki

**Affiliations:** 1 Department of Tissue Biochemistry, Graduate School of Medicine, Osaka University, Osaka, Japan; 2 Department of Tissue Biochemistry, Graduate School of Frontier Biosciences, Osaka University, Osaka, Japan; 3 Premium Research Institute for Human Metaverse Medicine (WPI-PRIMe), Osaka University, Osaka, Japan

**Keywords:** Articular cartilage, Chondrocytes, Induced pluripotent stem cells, Allogeneic transplantation, Immune response

## Abstract

**Background:**

Because of its poor intrinsic repair capacity, articular cartilage seldom heals when damaged.

**Main body:**

Regenerative treatment is expected for the treatment of articular cartilage damage, and allogeneic chondrocytes or cartilage have an advantage over autologous chondrocytes, which are limited in number. However, the presence or absence of an immune response has not been analyzed and remains controversial. Allogeneic-induced pluripotent stem cell (iPSC)–derived cartilage, a new resource for cartilage regeneration, reportedly survived and integrated with native cartilage after transplantation into chondral defects in knee joints without immune rejection in a recent primate model. Here, we review and discuss the immunogenicity of chondrocytes and the efficacy of allogeneic cartilage transplantation, including iPSC-derived cartilage.

**Short conclusion:**

Allogeneic iPSC-derived cartilage transplantation, a new therapeutic option, could be a good indication for chondral defects, and the development of translational medical technology for articular cartilage damage is expected.

## Background

Articular cartilage covers the ends of bones and serves as a lubricant to ensure smooth joint movements. Articular cartilage consists of chondrocytes embedded in an abundant extracellular matrix (ECM), which is composed of type II, IX, and XI collagen molecules and proteoglycans. The ECM enables the mechanical functions of the cartilage. Cartilage has a limited regenerative capacity, and its damage tends to result in degenerative conditions, impairing joint function. Microfractures and autologous osteochondral transplantation have been used for relatively small defects (less than 2–3 cm^2^) as a treatment for articular cartilage damage [1, 2]. Healthy articular cartilage is called hyaline cartilage and is composed of hyaline cartilage rich in collagen II and proteoglycans. However, when cartilage ECM is lost due to injury or degeneration, hyaline cartilage degenerates into fragile fibrocartilage rich in collagen I, compromising its function as an articular cartilage. During cartilage repair after injury, fibrocartilage is formed due to aberrant collagen expression. Fibrocartilage is the result of cartilage fibrosis, and in many cases, repaired fibrocartilage, which lacks the original function, shows inferior mechanical properties, and even worsens osteoarthritis symptoms [3]. Microfracture induces progenitor cells from the bone marrow to repair the defect; however, the tissue repaired by microfracture consists of fibrocartilage which does not have the mechanical robustness of hyaline cartilage and is therefore vulnerable to mechanical forces of the joint. In one case series study, microfracture showed good short-term results in the treatment of small cartilage defects, but the deterioration of results began 18 months postoperatively and was most pronounced in the International Cartilage Repair Society (ICRS)-score [4]. Clinical outcomes of microfracture have tended to either plateau or deteriorate at longer follow-ups, raising concerns about long-term results [5]. Autologous osteochondral transplantation carries the risk of donor-site morbidity.

The transplantation of autologous cultured chondrocytes is the most commonly used cell-based therapy for treating human cartilage defects. However, chondrocytes lose their chondrocyte nature after expansion in culture, and most of the repaired tissue is fibrocartilage tissue [6–8]. As only a limited number of autologous chondrocytes are prepared, repair is thought to occur through the trophic effect of growth factors and other factors produced by transplanted cells that stimulate host cells. This treatment is a two-stage procedure and carries the risk of donor site morbidity. Chondrocytes have been shown to have limited major histocompatibility complex (MHC) expression and immunosuppressive potential in vitro [9], and allogeneic chondrocyte transplantation has also been studied. However, their in vivo immunogenicity remains controversial [10]. One of the other major cell sources for cartilage repair is mesenchymal stem cells (MSCs), which can be obtained from bone marrow, adipose tissue, and synovium. MSCs can differentiate into chondrocytes and can also achieve therapeutic effects through paracrine effects. Furthermore, MSCs are involved in the cartilage repair process by modulating the immune response when damaged cartilage is exposed to an inflammatory environment. Recent reviews have reported that functional heterogeneity of MSCs among donors, tissues, and MSC subpopulations leads to differences in cartilage repair capacity [11].

Cartilage tissue is considered immune-privileged because it is avascular and chondrocytes are surrounded by ECM [9, 12]. The ECM inhibits immune cells from contacting chondrocytes, thus avoiding immunological reactions, even under allogeneic conditions. Allogeneic cartilage has been transplanted in clinical practice without human leukocyte antigen (HLA) matching or the use of immunosuppressive agents [13–15]. However, there are risks of donor shortage, heterogeneity in quality, and disease transmission. Recently, clinical trials using allogeneic human iPSCs have been conducted as a new regenerative medicine [16, 17]. iPSCs have unique pluripotency and self-renewal properties shared with embryonic stem cells (ESCs). iPSCs are created by introducing reprogramming factors into somatic cells, such as skin or blood cells, whereas ESCs are acquired from the internal cell mass of embryos. Human iPSCs do not bear the ethical issues associated with the sacrifice of embryos associated with human ESCs. iPSCs are a promising resource for the regenerative treatment of diseased or damaged organs and tissues, including articular cartilage damage, and the generation of iPSC-derived cartilage composed of chondrocytes and ECM has already been reported [18–20]. The self-renewal ability of iPS cells enables an unlimited supply of allogeneic iPSC-derived cartilage, solving the problems of allogeneic cartilage, such as the scarcity of donors and variations in cartilage quality among donors. Thus, allogeneic iPSC-derived cartilage transplantation may be a viable treatment option for articular cartilage damage. However, there have been reports of rejection after allogeneic chondrocyte transplantation [21–24], and it remains controversial whether transplanted allogeneic cartilage can cause an immune response. In this review, we summarize the immune response and efficacy of allogeneic cartilage transplantation for articular cartilage injury and discuss the recent advances in allogeneic iPSC-derived cartilage transplantation.

### Immunogenicity of chondrocytes in vitro

**Fig. 1:**
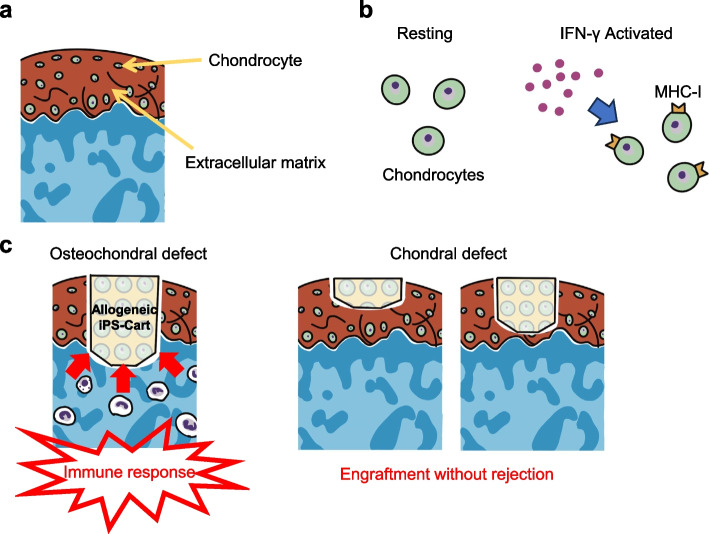
Limited immunogenicity of articular cartilage. **a** Histological image of articular cartilage. ECM inhibits contact of immune cells with chondrocytes. **b** Expression of MHC-I molecules on chondrocytes. Chondrocytes stimulated with IFN-γ show increased expression of MHC-I, suggesting that in the absence of ECM, they can become immunogenic under inflammatory conditions. **c** Immune response in allogeneic iPSC-derived cartilage transplantation. In osteochondral defects, T cells are observed around the graft. On the other hand, the graft is engrafted without immune response in chondral defects

Articular cartilage is generally considered immune-privileged because of its avascularity and because chondrocytes are embedded in the ECM (Fig. 1a). Previous in vitro studies have reported on the immunogenicity of chondrocytes prepared by the digestion of the ECM with collagenase or other agents. They reported that the co-culture of chondrocytes with allogeneic lymphocytes did not promote lymphocyte proliferation [9, 25–27]. Juvenile chondrocytes are less immunogenic than adult chondrocytes because of their lower HLA expression and have a stronger anabolic effect on ECM formation [9, 26, 28]. In addition, the immunosuppressive potential of chondrocytes has been reported, and the chondrocyte expression of B7 family members (B7-H1, B7-DC, B7-H2, B7-H3, and B7-H4), which act as inhibitory signals to T cells, chondromodulin-I, a T cell growth inhibitor, and indoleamine 2,3-dioxygenase, a mediator of immune evasion, has been suggested as a mechanism of immunosuppression [9]. These results suggest that chondrocytes are immune privileged, at least in vitro. However, juvenile chondrocytes stimulated with recombinant human interferon γ (IFNγ) show an increased expression of MHC Class I (HLA-ABC) (Fig. 1b); therefore, they can become immunogenic under inflammatory conditions, such as osteoarthritis [9].

### Immunogenicity of chondrocytes in vivo

There have been numerous reports of allogeneic chondrocyte transplants that are not rejected in vivo in animal models of knee cartilage defects [29–36]. However, some studies have observed certain immune responses, such as lymphocyte and macrophage aggregation [21–24, 37]; therefore, allogeneic chondrocyte transplantation in vivo is controversial. A combination of various factors, including differences in the animal species used, the method of preparation of chondrocytes for transplantation, and even the technique of defect preparation, may be responsible for these conflicting results [10]; however, the details of the immune response in allogeneic transplantation of chondrocytes have not been elucidated. Cartilage injuries are classified as osteochondral or chondral defects. One major difference between the two types of defects is that, in osteochondral defects, the graft is exposed to blood flow from the bone marrow. Blood flow can significantly affect immune responses. It has been reported that cartilage formed by the transplantation of allogeneic chondrocytes into articular cartilage defects in rats was infiltrated by immune cells migrating from the bone marrow, but not on the surface of transplants facing the joint cavity. This result suggests that the immune response occurs via the bone marrow and not the joint cavity [38].

Cellular infiltration involved in the rejection of cartilage formed by intramuscular allogeneic chondrocyte transplantation has been evaluated immunohistochemically in rats [39]. It has been suggested that activated monocytes, macrophages, and chondrocytes are involved in the lysis of the cartilage matrix during the rejection process and that the death of transplanted chondrocytes is mediated by infiltrating cytotoxic lymphocytes. There are no detailed reports on the immune responses of grafts to cartilage defects, and it is uncertain whether a similar process follows.

### Allogeneic cartilage grafts in animal models

Studies of particulate juvenile allograft cartilage (PJAC) have been widely studied in allogeneic cartilage tissue transplantation. PJAC is composed of minced live cartilage allografts from juvenile donors containing chondrocytes within their native extracellular matrix. Juvenile cartilage is considered less immunogenic, and minced cartilage allows chondrocytes to diffuse out of the ECM to form new hyaline-like cartilage [9, 26, 28]. Juvenile chondrocytes show excellent ECM production capacity in vitro [26, 28], and increased fragmentation significantly enhances ECM production [40]. A study in a rabbit model showed that minced cartilage grafts embedded in atelocollagen gel repaired osteochondral defects to the same extent as autologous chondrocyte implantation (ACI) [41], whereas another study reported that PJAC repaired osteochondral defects with hyaline cartilage-like tissue and showed significantly higher modified O'Driscoll scores than controls [42]. Studies in pigs have shown that PJAC transplants repair full-thickness cartilage defects as well as autologous cartilage chips [43] and that the transplanted cells survive for at least 3 months [44]. In animal studies, including two osteochondral defect models and two full-thickness cartilage defect models, allogeneic cartilage transplantation resulted in good cartilage repair; however, the immune response was not investigated (Table 1).

**Table 1:** Animal studies in allogeneic cartilage transplantation for cartilage defects of knee joints

References	Species	Location	Defect type	Follow-up	Grafted tissue	Outcome	Immune response
Bonasia et al. (2016) [42]	Rabbit	Trochlea	Osteochondral defect	3 and 6 months	Juvenile cartilage fragments	Histologically, PJAC performed better than control.	None mentioned.
Ao et al.(2019) [43]	Minipig	Trochlea	Full-thickness cartilage defect	1, 3, and 6 months	PJAC	No statistical difference in repair effect between PJAC and ACC at 6 months.	None mentioned.
Matsushita et al.(2019) [41]	Rabbit	Trochlea	Osteochondral defect	4, 12, and 24 weeks	Minced cartilage in atelocollagen gel	Implantation of minced cartilage embedded in atelocollagen gel showed good cartilage repair equivalent to ACI.	None mentioned.
Zhang et al. (2022) [44]	Minipig	Trochlea	Full-thickness cartilage defect	1 and 3 months	PJAC	SOX9 expression was stronger in the PJAC than ACC group at 3 months. Transplanted cells survived at least 3 months.	None mentioned.
Okutani et al. (2022) [45]	Cynomolgus monkey	Trochlea	Osteochondral defect	4 weeks	cyiPSC-derived cartilage	cyiPSC-derived cartilage survived and was not rejected.	Accumulation of lymphocytes in bone marrow.
Abe et al. (2023) [46]	Cynomolgus monkey	Trochlea	Osteochondral defect Chondral defect	4 and 17 weeks	cyiPSC-derived cartilage	cyiPSC-derived cartilage survived and integrated with host native articular cartilage in chondral defects.	Accumulation of lymphocytes in osteochondral defects. None in chondral defects.

### Clinical studies in PJAC transplantation

Particulated juvenile allograft cartilage products (DeNovo Natural Tissue [NT], manufactured by Zimmer Biomet) have been available since 2007. By 2015, more than 8700 patients had been treated with DeNovo NT [47]. This product is a minced live cartilage graft from a juvenile donor that contains cartilage cells and surrounding ECM. Minced cartilage is expected to further promote ECM formation and repair by the graft itself. However, the data on mid- and long-term clinical outcomes are lacking. Several case series have reported short-term (2–3 years) improvements in clinical scores (KOOS, IKDC, VAS, etc.) and moderate to good filling of defects on post-transplantation MRI findings [13–15, 48–50]. Complications, such as graft hypertrophy, delamination, and displacement have been reported. However, no studies have evaluated the immune response or rejection. The evidence of PJAC transplantation for cartilage injuries remains insufficient; however, short-term studies have shown promising results (Table 2).

**Table 2:** Clinical studies of PJAC transplantation

References	Study type	Location	Sample size	Lesion size	Follow-up	Outcome measure	Outcome	Complication related to grafts
Tompkins et al.(2013) [48]	Case series	Patella	15 knees	2.4 ± 1.2 cm^2^	28.8 months	KOOS, IKDC, Kujara, Tegner, VAS, MRI	Mean fill of defect at follow-up was 89%.	2 lesions: graft hypertrophy 1 lesion: complete graft failure/delamination
Farr et al. (2014) [13]	Case series	Femoral condyle or Trochlea	29 lesions	2.7 ± 0.8 cm^2^	24 months	KOOS, IKDC, VAS, MRI	Improved in KOOS, IKDC, and VAS; T2-weighted scores were returning to a level approximating that of normal articular cartilage by 2 years.	2 lesions: partial graft failure/delamination 1 lesion: partially filled defect
Buckwalter et al. (2014) [49]	Case series	Patella	13 patients	2.3 ± 1.8 cm^2^	8.2 months	KOOS, WOMAC	Improved in KOOS overall.	none
Grawe et al. (2017) [50]	Case series	Patella	45 patients	2.1 ± 1.2 cm^2^	6,12, and 24 months	MRI	85% of patients at 12mo displayed good to moderate fill. Demonstrated progressive graft maturation over time by imaging.	1 patient: graft displacement 2 patients: graft hypertrophy
Wang et al. (2018) [14]	Case series	Patella or trochlea	30 lesions	2.1 ± 1.2 cm^2^	3.8 years	IKDC, KOS-ADL, MAS, MRI	Improved in IKDC and KOS-ADL, no change in MAS. 69% of patients had a majority lesion fill.	none
Dawkins et al. (2022) [15]	Case series	Patella or trochlea	36 knees	2 cm^2^	33.8 months	Return to sport rate, MRI	Return to sports rate was 100%. 78% of patients had a majority defect fill.	1 knee: Tissue delamination 1 knee: Full-thickness graft fissuring

### Immunogenicity in iPSC-derived cartilage

In a study on iPSC-derived chondrocytes, a new resource for cartilage regeneration, iPSC-derived chondrocytes showed limited HLA expression and did not induce lymphocyte proliferation in a mixed lymphocyte assay when co-cultured with allogeneic peripheral blood mononuclear cells [51]. Treatment with IFNγ induces the expression of major histocompatibility complex (MHC) class I, but not MHC class II, in iPSC-derived chondrocytes, being similar to juvenile chondrocytes and potentially immunogenic under inflammatory conditions [9, 51]. Thus, the immune response of iPSC-derived chondrocytes and juvenile chondrocytes in an in vivo inflammatory environment such as osteoarthritis should be further evaluated. These results collectively suggest that iPSC-derived chondrocytes have similar immunogenic properties to those of juvenile chondrocytes in vitro, so allogeneic iPSC-derived cartilage transplantation can be performed without the use of immunosuppressive agents as in PJAC transplantation.

Allogeneic transplantation of iPSC-derived cartilage was performed in a primate model by mismatching the MHC, which is structurally similar to HLA, to verify the immune response in vivo. Cartilages generated from cynomolgus monkey iPS cells (cyiPSCs) were transplanted into chondral or osteochondral defects in the femoral trochlea of MHC-mismatched monkeys without the use of immunosuppressive drugs. Four weeks after allogeneic transplantation, although the graft remained intact, an accumulation of CD3-positive T cells was observed around the graft in osteochondral defects. In contrast, in chondral defects, the graft is engrafted without lymphocyte accumulation [45, 46]. It has been suggested that in chondral defects, the immune response is suppressed because there is no contact between the graft and bone marrow (Fig. 1c).

### Allogeneic iPSC-derived cartilage transplantation for chondral defects

Allogeneic transplantation of cyiPSC-derived cartilage for chondral defects showed that the cyiPSC-derived cartilage was engrafted and contributed directly to hyaline cartilage-rich repair 4 months after transplantation [46].

Integration of the graft and host cartilage is essential for successful tissue replacement as it provides stable biological fixation and load distribution as well as adequate mechanotransduction necessary to maintain homeostasis [7]. However, cartilage-to-cartilage integration is exceedingly difficult to achieve because of the low metabolism of cartilage and the high density of the anti-adhesive ECM [52, 53]. Allogeneic cyiPSC-derived cartilage transplanted into chondral defects was well integrated with the host side cartilage, suggesting that stable biological fixation was achieved [46]. Human iPSC-derived cartilage has shown capacity for integration, and fibroblast growth factor (FGF) signals are involved in this integration [54]. RNA sequencing analysis showed a higher expression of *FGF18* in the perichondrium-like membrane of human iPSC-derived cartilage. The addition of FGF18 promoted the integration of cartilages, whereas the addition of FGFR inhibitors inhibited it. These suggested that FGF18 secreted from the perichondrium-like membrane is involved in the integration of the human iPSC-derived cartilage [54].

Articular cartilage is a layered tissue that expresses proteoglycan 4 (PRG4), which functions as a lubricant in the superficial layer [55–57]. Post-transplantation, cyiPSC-derived cartilage showed high expression of PRG4 in the superficial layer. These results suggest that cyiPSC-derived cartilage acquires lubricating ability after transplantation and functions as articular cartilage [46]. One of the major differences between pre- and post-transplantation is that the graft is subjected to shear forces associated with knee joint motion in vivo after transplantation. Shear forces in vivo have been reported to stimulate PRG4 expression in chondrocytes via cyclic adenosine monophosphate (cAMP) signaling [58], and the expression of PRG4 in cyiPSC-derived cartilage after transplantation has been suggested to be associated with shear forces. Furthermore, salt-inducible kinase 3 (SIK3) has been suggested to be involved in PRG4 expression after transplantation [46]. SIKs inhibit nuclear translocation of cAMP response element binding protein (CREB)-regulated transcription coactivator (CRTC) by phosphorylating, thereby repressing CREB activation and gene transcription. Among the members of the SIK family, SIK3 functions primarily in chondrocytes. A recent study demonstrated that deletion of Sik3 further increased shear stress-induced *Prg4* expression in mouse chondrocytes, suggesting that Sik3 negatively regulates *Prg4* expression [46].

### Suppression of immune response during allogeneic iPSC-derived cartilage transplantation into osteochondral defects

Allogeneic cyiPSC-derived cartilage transplantation for osteochondral defects showed that lymphocytes clustered around the graft 4 weeks postoperatively [45, 46]; however, the graft itself remained, suggesting a temporary immune response rather than complete immune rejection. Because chondrocytes express molecules that transduce inhibitory signals to T cells [9], these molecular mechanisms may contribute to the survival of allogeneic cyiPSC-derived cartilage in osteochondral defects.

The results of allogeneic iPSC-derived cartilage transplantation in a primate model with an immune system similar to that of humans suggested that chondral defects are a better indication for allogeneic iPSC-derived cartilage transplantation than osteochondral defects. Regarding osteochondral defects, an immune response has been observed in cases of MHC mismatches, which remains a long-term concern. One solution to prevent immune reactions is to minimize rejection by matching the HLA types of the donor and host cells. iPSC lines are established from donors whose major HLA types are homozygotes [59, 60]. These HLA-homo iPSC-derived products matched recipients with an identical set of HLA types in one allele. It is estimated that an iPSC line homozygous for the most frequent HLA types in the Japanese population would match 17% of the Japanese population. Therefore, preparing HLA-type homozygous iPSCs could minimize the influence of immune rejection [59, 60]. Another solution is to genome-edit iPS cells by using techniques, such as the CRISPR/Cas9 system [61]. It has been reported that *B2M*^−/−^ cyiPSC-derived cartilage knocking out β2 microglobulin, which does not express MHC class I, was transplanted into osteochondral defects and showed immune response by NK cells [48]. This result is consistent with a previous finding that natural killer (NK) cells recognize and eliminate cells that fail to express MHC class I molecules [62]. Recent studies have established that human iPSCs lack HLA class I and II molecules and suppress NK cell attack by HLA-E transduction, CD47 overexpression, or PVR knockout [63–67]. Currently, the HLA genome-edited iPS stock is available for research use [67]. The use of cartilage tissue differentiated from HLA genome-edited iPS cells can suppress the immune response to allogeneic transplantation for osteochondral defects.

### Safety and costs of allogeneic iPSC-derived cartilage transplantation

Transplantation of autologous iPSC-derived grafts is desirable in terms of avoiding immune reactions and minimizing the risk of spreading communicable viral infections; however, cost and manufacturing lead time is not practicable for commercialization. When the world’s first autologous transplant of iPSC-derived retinal pigment epithelial (RPE) cells was performed, the patient had to wait for more than 10 months from harvesting skin tissue to RPE sheet transplantation, which cost nearly 100 million yen [60, 68]. On the other hand, allogeneic transplantation approaches can reduce the cost and time of the iPSC manufacturing process. In the case of allogeneic iPSC-derived RPE sheet transplantation using cells from iPSC stock, the preoperative waiting period was approximately 1 month, and the total cost per patient was about one-fifth of autologous transplantation [60]. Similarly, the use of allogeneic iPSC stock reduces cost and time in iPSC-derived cartilage transplantation. One of the particular safety risks of iPSC-derived therapies, including iPSC-derived cartilage transplantation, is the concern of tumorigenicity because possible contamination of undifferentiated iPS cells can give rise to teratoma. The reprogramming process of the iPSC and the long culture time for cartilage differentiation could increase the potential for malignancy. The risk of tumorigenicity has been thoroughly evaluated in preclinical tests including in vitro expression analysis of iPS cell markers to detect contamination of iPS cells in the cartilage and in vivo tumorigenicity testing in which iPS cell-derived cartilage is transplanted into immunodeficient rats orthotopically for life-long observation. The evaluation of the tumorigenic risk of iPSC-derived cartilage using HeLa cells as the reference control suggested that the potential benefit of the therapy outweighs the risk of tumor formation and the clinical application of iPSC-derived cartilage in the knee joint was considered acceptable [69].

## Conclusions

**Fig. 2:**
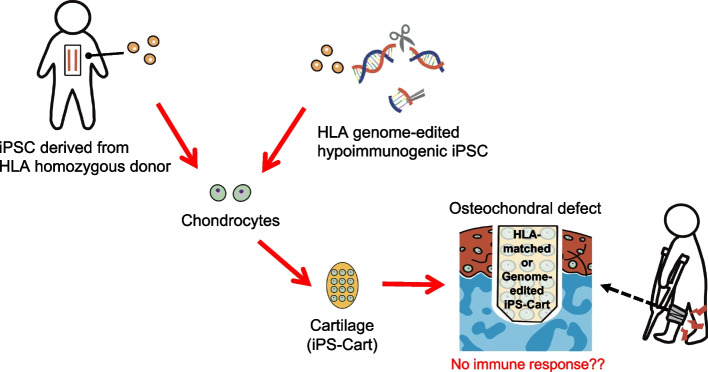
Solution to prevent immune response by iPSC-derived cartilage in osteochondral defects. HLA-matched or HLA genome-edited hypoimmunogenic iPSC lines can suppress the immune response in osteochondral defects

Chondrocyte and cartilage transplantation are treatment options for cartilage defects. Although chondrocytes have been shown to be hypoimmunogenic in vitro, allogeneic chondrocyte transplantation is controversial due to reports of immunoreactivity in vivo. In contrast, allogeneic cartilage transplantation, as performed in clinical practice, tends to show promising results in the short term; however, the evidence is insufficient. There are two types of cartilage defects, osteochondral and chondral defects, which may differ in their immune responses during allogeneic transplantation. The immune response to chondrocytes can be triggered by contact with the bone marrow. Although the effect of immune reactions on clinical outcomes in osteochondral defects has not yet been determined, immune reactions should be controlled to ensure good repair. Allogeneic iPSC-derived cartilage transplantation, a new therapeutic option, could be a good indication for chondral defects without an immune response. HLA type matching or iPSC lines in which HLA genes are edited can provide a solution to suppress the immune response in osteochondral defects (Fig. 2).

## Data Availability

All the original data are available upon request from the authors.
